# Innovation in mental health services: what are the key components of success?

**DOI:** 10.1186/1748-5908-6-120

**Published:** 2011-10-26

**Authors:** Helen Brooks, David Pilgrim, Anne Rogers

**Affiliations:** 1 Health Sciences, Primary Care, Community Based Medicine, University of Manchester, Manchester, UK; 2 School of Social Work, University of Central Lancashire, Preston, UK; 3 National Institute for Health Research, School for Primary Care Research, Community Based Medicine, University of Manchester, Manchester, UK

## Abstract

**Background:**

Service development innovation in health technology and practice is viewed as a pressing need within the field of mental health yet is relatively poorly understood. Macro-level theories have been criticised for their limited explanatory power and they may not be appropriate for understanding local and fine-grained uncertainties of services and barriers to the sustainability of change. This study aimed to identify contextual influences inhibiting or promoting the acceptance and integration of innovations in mental health services in both National Health Service (NHS) and community settings.

**Methods:**

A comparative study using qualitative and case study data collection methods, including semi-structured interviews with key stakeholders and follow-up telephone interviews over a one-year period. The analysis was informed by learning organisation theory. Drawn from 11 mental health innovation projects within community, voluntary and NHS settings, 65 participants were recruited including service users, commissioners, health and non-health professionals, managers, and caregivers. The methods deployed in this evaluation focused on process-outcome links within and between the 11 projects.

**Results:**

Key barriers to innovation included resistance from corporate departments and middle management, complexity of the innovation, and the availability and access to resources on a prospective basis within the host organisation. The results informed the construction of a proposed model of innovation implementation within mental health services. The main components of which are context, process, and outcomes.

**Conclusions:**

The study produced a model of conducive and impeding factors drawn from the composite picture of 11 innovative mental health projects, and this is discussed in light of relevant literature. The model provides a rich agenda to consider for services wanting to innovate or adopt innovations from elsewhere. The evaluation suggested the importance of studying innovation with a focus on context, process, and outcomes.

## Background

Health service providers are increasingly seeking new ways of working to improve quality by increasing cost-effectiveness and encouraging innovation in technologies and practices. The implementation of these innovations and improvements has also become an important focus for current healthcare research. Whilst the translational gap between novel innovations and their implementation has been identified as an area for particular attention [1,2], implementation processes are still not well understood in the field of mental health. Here, we examine innovations in mental health services in order to progress an understanding of the barriers and enabling factors associated with implementation.

Theories of innovation applied to healthcare settings have tended to focus on a 'whole systems' approach to mapping the potential for the successful implementation of innovative practices and the ability of organizations to create, innovate, and deploy new systems of practice [3]. Thus, unsurprisingly, innovation research and analysis has highlighted the dynamics of diffusion, organizational performance, and integration.

However, the complexity of abstracted levels makes interpretation difficult to apply in real life settings [4], and the focus on the organizational level has failed to produce evidence of effectiveness. One study found that despite rigorous evaluations, the evidence for strategies to improve organisational innovation is limited and that, 'for no strategy can the effects be predicted with high certainty' [5]. A more recent review suggested that current available evidence does not identify any effective, generalizable strategies for changing organizational culture [6].

One of the problems seems to be that macro-level theories about implementation struggle with accounting for context and action at different levels. Thus, evaluations with criteria that, at the outset, focus on interactional processes and developments in context may yield better insights. We know that in the mental health field sensitivity to local circumstances are revealing, particularly when considering introducing new and complex interventions into open and community settings [7]. In this paper, we examine the conditions and environments under which interventions emerge and become workable in context, and the challenge of transferring learning about these to new sites of implementation.

### Innovation in mental health

Healthcare innovation, infrastructure, and science and technology are identified as important in service development within mental health services [8]. For example, in the United Kingdom, the implementation of the first National Service Framework (NSF) introduced by the Labour Government when they came into power in 1997 outlined a number of policy assumptions about service improvement [9].

However, mental health services historically have been marginalised and neglected, implying the need for the introduction of radical innovation [8]. We know that countries with the best performance in the field of mental health (in terms of publication of scientific papers and production of patents related to mental health) have the best mental health infrastructure and are also ranked first in science and technology in this area. Countries with the worst performance in the field of mental health also have the worst mental health infrastructure and are in the worst position in science and technology. Factors such as the unexpected convergence of national policies, local structures, and de-institutionalisation and associated politics have also created potential spaces and opportunities for a process of change [10].

Whilst specific aspects of mental health treatments such as medications are often identified as problematic and needing reform [11], mental health innovation is rarely the topic of focus in and of itself. Barriers to innovation even if they are evidence-based suggest that understanding the organizational and policy context at a local level is important. For example, a randomized controlled trial (RCT) demonstrated that peer workers were effective at connecting people with mental health problems with services [12]. However, policy makers considered the initiative a failure. The authors considered that this arose because the competing political, organizational, and evaluative demands produced a disjuncture between political expectations and programmatic capacities. In this case, peer specialists were not able to help their clients in ways seen as directly relevant to policy makers [12].

Similarly, in a study introducing innovation for homeless people with mental health problems, the mode of presentation, use of an outside agency, and the questioned uniqueness of the new practice were found to be as important as the intervention itself [13]. Most importantly, as Proctor *et al*. suggest in their paper on mental health implementation in 2008 there is a paucity of evidence that innovations are adopted or successfully implemented in community settings in an appropriate and relevant way. The authors suggested four levels of change (larger system, environment, organization group, and team) for assessing performance improvement, and these levels have helped interpret the data presented in this paper [14]. Proctor *et al*. highlight individual assumptions about change which are important to consider including [14]: reimbursement, legal and regulatory policies; cooperation, co-ordination, and shared knowledge; structure and strategy; and knowledge, skill, and expertise.

With the above background in mind, this paper now moves to consider innovation implementation by examining projects at a contextual level and examines the attempts to offer innovations in mental health services with reference to existing relevant literature.

### External organisational impetus: NESTA's role in evaluating innovations in mental health services

Based on the outcome of an evaluation of eleven innovation projects commissioned by the National Endowment for Science Technology and the Arts (NESTA), we attempt to use the central concepts and themes that emerge in the context of existing literature to produce a tentative theoretical model of innovation. The organisational focus of NESTA is on supporting innovation in Britain in the public, private, and 'third' sectors. The latter now includes voluntary organisations and 'social enterprises' (small businesses with state funding to pursue socially valued goals).

In 2006, the NESTA conducted an exercise to establish UK priorities about social innovations and how they might be stimulated and supported. In November 2006, mental health emerged as one of these priorities for the funding body.

The funding scheme was launched in March 2007, and partners provided support with promoting the fund through their networks. In all, £500,000 was released to spend across the projects and to fund some management support to them. The call for bids was released with a set of criteria and it placed an emphasis on projects demonstrating: the innovative nature of the project; multi and interdisciplinary working; use of arts in the mental health field; and service-user engagement.

The call generated over 500 applications, which varied greatly in terms of content, quality, and the type of organisation applying. The applications process resulted in 11 projects being selected to obtain funding. In 2008, another call was then made for research organisations to evaluate the 11 projects. The authors of this paper were appointed to this role. In addition to this overview evaluation, some of the individual projects had built in additional local audits or evaluations, which were fully accessible to the authors.

### Summary of the eleven projects

The projects funded varied in a number of ways. Some were extensions of current projects whereas others were completely fresh in vision and intent. They also varied in size and in the amount of money offered to them for support. Some were inside and others outside the National Health Service (NHS). Some focused on offering people with mental health problems ordinary activities and others on improving the quality of mental health services. Table 1 outlines the individual projects. The localities and names of the projects are made anonymous here for public purposes but they are public on the NESTA website along with a report covering material in this paper.

**Table 1:** Description of individual projects

	Working with homeless people with mental health problems	Computer skills for people with dementia	Educating about the subtle abuse of vulnerable people	Taking theatre skills into a secure mental health unit	A mental health self-help kit	The therapeutic use of animation with children	Web-based feedback from service users	Improving service provider communic-ation skills	Working with traumatised refugees and asylum -seekers	User involvement in mental health worker training	Involving users in designing inpatient environments
**Brief description of activities**	Provision of user defined arts activities to homeless service users	Provision of IT training to service users with dementia	Artist and service user collaboration to produce an informative DVD about the subtle abuse	Provision of arts activities to excluded groups *e.g.*, those within secure mental health units	The production of a prototype mental health self-help kit	This project explored the therapeutic use of animation with vulnerable children	Extension of existing web based feedback system into mental health services	Communication skills workshops and an interactive DVD for use with health professionals	NHS Trust collaboration with city farm providing gardening activities combined with therapy	Buddy scheme between service users and trainee mental health workers	Production of a prototype board game to engage services in the design of inpatient environments

**Relation to statutory services**	External	External	External	External	External	External	External	Internal	Internal	Internal	Internal

**Changes to the hosting of the project**	No	No	No	No	No	Yes - moved outside statutory services.	No	No	No	No	Yes - Trust reorganisation

**Level of development prior to the NESTA grant**	New	New	New	Expansion	Existing	New project	Expansion	Expansion	Expansion	Existing	New

**Was the output of the project a product or an activity?**	Activity and products	Activity and product	Activity and product	Activity	Product	Activity and product	Product	Activity and product	Activity	Activity and product	Product

**Ethos of the project**											
**Therapy low/high**	Low	Low	Low	Low	High	High	Low	Low	High	Low	Low
**USER Inclusion low/high**	High	High	High	High	High	High	High	High	High	High	High

## Methods

### Producing the implementation model -- a realistic evaluation of the projects

In order to generate a model of innovation implementation within mental health services, the evaluation aimed to provide a rich picture of the 11 projects and to understand the extent of success of each project. This was achieved by eliciting the views of key stakeholders involved in the project, examining project documentation, and the use of local evaluations where they were available. In this article, it is those cross-project lessons that will be reported in the form of a theoretical model along with tables of key findings.

The methods deployed for the purpose of this study focused particularly on process-outcome links within and between the 11 projects examined. We used the principles of realistic evaluation by Pawson and Tilley [15]. They emphasise an understanding of mechanisms operating in particular contexts that create outcomes. Particular attention is then drawn to what is working for whom according to the stakeholders involved in the project. In our case, this approach was applied to each of the 11 projects, generating conclusions about each but then permitting comparisons to be made across the piece. Data were examined independently by the authors (HB and DP). Initially significant words, phrases, and paragraphs were noted. Lists of emerging themes were then drawn up for each project, and the authors met to reach agreement on a list of cross-project lessons. This list was then compared across projects and a final table of the occurrence of themes was produced after extensive discussion (Tables 2 and 3).

**Table 2:** Conducive conditions for innovation cited by interviewees

CONDUCIVE FACTORS	OCCURRENCE % (n)
**Context**	

The skills, knowledge and experience of the project team, especially the project champion	91% (10)

Supportive team	73% (8)

The project was aligned to the core business of the host organisation	73% (8)

The project champion's position within the system	55% (6)

Independent organisation which was external to statutory services	55% (6)

A team working towards a common goal	45% (5)

The provision of a safe environment for service users	45% (5)

Sustained management 'buy-in' or support at all levels	45% (5)

The small size of the organisation and a flat team hierarchy	45% (5)

The forward looking/innovative nature of the host organisation	27% (3)

Strong networks, *e.g*., links with local and voluntary organisations	27% (3)

The project builds on the work of an existing project	27% (3)

Support for the project from national policy drivers	27% (3)

Organisational control is devolved to hosts or project champion	27% (3)

Effective partnership working (trust and respect developed)	18% (2)

**Process-outcome**	

The assertive and committed actions of the project champion.	100% (11)

The positive role of service users (when service user involvement is active)	100% (11)

The support from the funding body (financial and non-financial)	100% (11)

External validation from funding body through provision of funding, national policy priorities, organisational vision etc	73% (8)

The positive role of staff within, or outside of, the host organisation	73% (8)

Flexibility of delivery	55% (6)

A constellation of supportive individuals within, and outside of, statutory services	45% (5)

Open and direct channels of communication.	45% (5)

Full documentation of project activity (including contact with authors)	45% (5)

The project was not focussed on therapy per se but encourages social interaction and provides access to future activity	45% (5)

Power differences reduced between service users and providers	36% (4)

The versatility and scope of the project	36% (4)

The value and strength of original idea	36% (4)

The strength of the intellectual input into the project	27% (3)

Processes for embedding and link with other internal systems (*e.g*., curriculum development)	27% (3)

Structure/stability of sessions for service users	27% (3)

The use of non-traditional roles in delivering the project and allowing artistic freedom for service users	27% (3)

The long-term strategic outlook of project from the outset	18% (2)

Project allows therapeutic distance between service users and providers	18% (2)

The project arose from an identified need	18% (2)

A relationship of trust develops between artists and staff	18% (2)

**Table 3:** The factors that impeded intended innovation

IMPEDING FACTORS	OCCURRENCE
**Context**	

Resource limitations and high workload	6

Resistance from corporate departments (such as finance and HR)	5

Lack of stability in the system (restructuring and rapid policy changes)	7

Large size of organisation results in general inertia within the system	4

Hierarchy in host organisation or NHS (*e.g*., support at top not filtered down and middle managers, which protects the status quo)	4

Internal politics/bureaucracy (policies and procedures developed for routine practice not innovation)	4

A cultural norm of risk minimisation	2

Poor service user access to the internet	2

Lack of awareness of intellectual property, branding and business processes	2

High staff turnover	2

**Process/Outcome**	

Initial resistance from front line staff	7

Initial resistance from service users	4

The new role of entrepreneur in NHS (little history and knowledge of the role)	4

Initial low expectations amongst staff	4

Resistance from middle managers	3

Underestimation of costs	3

Professional jealousy/resource envy	3

Poor communication within host organisation (especially in the NHS)	3

Tensions between rhetoric at the top and action on the ground	2

Tension between artistic integrity and therapeutic benefit	2

Newness and complexity of idea (judged to be 'too left field')	2

Funding body had little influence on statutory services	2

The following six domains of information based up the realistic evaluation methodology were established, recorded, and reported on in the evaluation:

1. Conducive conditions: Given that a rationale for each programme was both proposed by local innovators and then endorsed by NESTA, the two parties assumed that the potential for service improvement was legitimate in principle. What evidence was established about the extent of conducive conditions for success in each of the 11 localities? What was learned about the extent to which those conditions enabled or constrained success?

2. Ontological depth: The *a priori *face rationale for each of the 11 projects led to them being commissioned. Our task was to try to understand the lived reality of each project from the perspective of the stakeholders involved.

3. Mechanisms: Information was elicited from the stakeholders about two sorts of mechanisms. The first refers to their understanding of the causal mechanisms that led to the problems they were trying to solve, counter, or ameliorate. The second refers to their understanding of the causal mechanisms they believed were involved in their restorative efforts in the latter regard. What were they trying to do to make improvements and what was their rationale for believing their actions would be effective?

4. Outcomes: The authors sought to understand the outcomes from a stakeholder perspective on a number of fronts. What outcomes were intended? What was achieved?

5. Context-mechanism-outcome patterns: Having generated a rich picture of each of the 11 projects, the next task in the evaluation was to identify patterns about the relationship between the context of the innovation, the mechanisms operating, and the outcomes evident. Did any patterns emerge between or across the projects that might illuminate the probability of spreading the innovation elsewhere?

6. Open systems: The realist rationale assumed that the evaluation was taking place in an open, not closed, system (thus distinguishing it from the laboratory or RCT style paradigms, where the investigator can control some of the conditions under scrutiny). The original context of each proposal may have changed because of new processes emerging in an open system. The evaluation would note the open system implications where they were relevant.

In the light of the above methodological rationale, the aims of the evaluation of the 11 projects were as follows:

### Aims

The aims of our evaluation were:

1. To provide a rich picture of the 11 projects.

2. To understand the extent of success of each project.

3. To draw conclusions from within and between the projects about potential success in new contexts.

Table 4 outlines the key methodological points or the essential elements guiding the analysis undertaken during the evaluation. This paper, however, reports on the cross-project lessons drawn from the 11 projects produced as a result of this analysis.

**Table 4:** Essential elements guiding the analysis undertaken

1. To describe each project in depth in terms of its operation in practice, intended or unintended outcomes, the extent of any success or failure, conducive or impeding conditions, and the causal mechanisms involved in generating and ameliorating the problem using stakeholder accounts.
2. To describe the unique features of each project and offer hypotheses about the extent these could be generalised to other contexts.
3. To establish the extent to which the conducive conditions in each local project may be different in other localities with different personnel.
4. To identify any patterns emerging between or across the projects, with a particular focus on spreading the lessons of innovation in mental health services.
5. To identify any evidence in each locality about open systems and the impact these may have on the success of the individual projects.

The evaluation was commissioned in 2008, ethics and research governance clearance was obtained in the spring of 2008, and the evaluation was completed by summer 2010.

## Results of the evaluation and discussion of the findings

**Figure 1:**
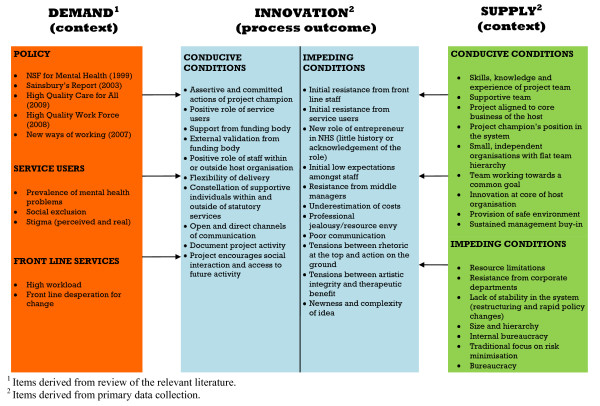
**Model of innovation derived from the data and relevant literature**.

The findings summarised in this article represent the main outcomes of the evaluation, which generated masses of rich data about the individual projects. By the end of the evaluation, 65 respondents had been interviewed for the purpose of the overview evaluation alone, and their views were placed in the context of the stated aims of the projects and were augmented by local evaluation findings where available. In this sense, where local evaluations were undertaken the findings were analysed from the perspective of the overview findings. There were no strongly contradictory findings identified from the local evaluations included within the study. For clarity, the findings are summarised in one main model (Figure 1) reflecting the methodological rationale noted earlier, which is then discussed in light of relevant literature. However, Tables 2 and 3 outline the main factors relating to conducive and impeding conditions for innovation.

The eleven projects were diverse in content and intention. Thus, any conclusions drawn about improvements of services for people with mental health problems across the piece are necessarily broad and schematic. At the same time, some of those lessons, because they are broad and emphasise open systems, may also offer insights about health and social care more broadly, and so are not limited to mental health. Taking both of these points into consideration, the findings will be discussed now in terms of the main features of the model (Figure 1) relating to context, structure, process, and outcome factors.

### Context

The context of innovation in the field of mental health includes, from the outset, the implicit notion that more can and should be done at odds with tradition. If the treatment of mental health problems in modern society, in its broad sense of societal responses and its narrow sense of effective ameliorative professional interventions, were already good enough, then either no innovation would be needed or it would be limited to simply offering 'more of the same.' Our shared current context suggests that neither would be warranted. Instead that context is characterised by other features, which encourage contradictory demands of risk taking and risk avoidance [16].

The need to reverse the social exclusion of people with mental health problems is now well recognised in both national and international policy priorities. Whilst both physical and psychological technologies have been developed to respond to 'mental disorder,' these interventions remain imperfect and at times have been a matter of controversy in professional and public circles, because of their contested cost-effectiveness and their particular iatrogenic risks and threats to civil liberty [16].

In this light, so much of the improvement in the professional care of people with mental health problems now focuses not on technical fixes ('therapy') but more on other matters, such as the local environment patient-centred care, opportunities for social inclusion and the enlargement of citizenship [17]. In particular, the emerging emphasis on 'recovery' for those with functional mental health problems of neurosis and psychosis (*i.e*., excluding dementia) brings together these points about social inclusion and consumerism. This policy and cultural context goes some way to explain the content of the projects chosen by the funding organisation.

Although consumerism and the general health policy shift towards 'patient-centredness' did not receive more attention (reflected in government policy and expectations to NHS managers from the Department of Health), as far as mental health problems are concerned this is not the whole story. The concern about threat to self and others, which are associated with these problems, means that services are also expected to minimise risk and avoid the adverse implications of risk taking. The culture of mental health services, which has been supported by the existence of dedicated mental health legislation, has therefore tended to emphasise paternalism not user-centred working. That paternalism is reflected in clinical norms about surveillance and control [18].

Innovation (or any failure to adhere to current policies and procedures) can be a systemic threat to norms and rules developed to ensure risk minimisation. This could explain why innovators were more frustrated when working inside the NHS than when they were external to the organisation. This contextual contradiction about risk taking and, on the other hand, to be risk averse, is continued in the next section.

### Structure

The relatively stable elements of a system (structure) relevant to understanding the findings are factors such as staff and resources. Clearly, having the right number of staff trained, in the right way, can enable innovations. Having a work force who are well trained and whose training is constantly updated is one of the key features of a 'learning organisation' [19]. Individual project champions were important, but so too were allies within the system with sufficient power to support their ambitions. Also, the rules that govern stability ('policies and procedures') were derived from past agreements about ways of working. These encourage routinisation, not innovation, and they determine the job descriptions of individuals and performance indicators for local organisations.

Routinisation means that when a problem is encountered in a system, the standard reaction is based on past and tested solutions, not on new ones that are untested. However, the emergent need for innovation *ipso facto *means that problems are not old and solvable ('tame problems') but new challenges not before encountered; Degrace and Hulet use the term 'wicked problems' [20]. Complex open systems (such as health and social care) may attempt to make improvements by conceiving all challenges as 'tame problems.' However, it soon becomes evident that many are 'wicked problems,' which can only be solved by new solutions and new ways of thinking (*i.e*., true innovation).

It is also the case that the opposite of structural stability (constant structural destabilisation) has been one reason that the British NHS in the past ten years has been unable to achieve the policy aspiration of becoming a 'learning organisation' [19]. Furthermore, the model supports studies that have shown that learning organisations are hard to develop in those organisations where management are unwilling to share power [21,22].

An example of this, which jeopardized the existence of one of the projects, relates to changes within the host organisation of one project based within the NHS. The project was nearly the fatal victim of NHS re-organisation, when the local service involved was taken out of one NHS Trust and placed in another. Because the decision to support the project was authorised by the senior managers of the original Trust, the 'new brush' of the adoptive Trust at first did not recognise its legitimacy or support its continuation. It was only after a period of lobbying from the project manager and her allies that the project was given permission to continue (as it happens, with much success). Thus, some degree of structural stability is required in order to provide innovations with the time to be tested out, learned from, and to retain any worthwhile improvements achieved.

### Process

Probably the strongest lesson learned across the 11 projects was in relation to project champions. The stereotypical features of those finding themselves to be 'hero-innovators' in systems were evident. Project champions were important and they were generally people who were determined and undeterred by any resistance encountered. They were risk takers and non-conformist in relation to role expectations. However, these individual qualities, although important for both establishing and sustaining projects were not sufficient. Projects emerged and survived as well because of the relationships they developed with others (usually sympathetic senior managers in the system). The management seniority included chief executive officer commitment, as well as the willingness of middle manager allies near to the project to solve problems of resistance as they arose.

This point about enabling relationships was also evident for those in organisational partnerships, whether that was between artists and staff in the NHS (such as the theatre project) or between organisations (for example the homelessness project). The circular truism that trust is important in any successful relationship proved to be evident when projects were going well. When problems arose, it was often when trust was weak or had broken down. For example, the near collapse of one of the projects noted above was because the newly adopted managers had no commitment to the project and so could not be trusted by others to ensure its survival. Their trust had to be gained afresh by lobbying from the project champion and her allies.

As the model seeks to demonstrate, support from management was important to the successful implementation of innovations. However, some projects did not have this support and the resistance from managers, particularly middle managers, could be severely detrimental to implementation. This resistance to innovation from managers is not a new consideration in the innovation literature. Vilela Chaves and Moro demonstrated that prevailing models and resources within services prevent managers from pursuing radical innovations and that those with restrictive views can adopt a range of rejection strategies towards any 'disruptive' innovations [8]. These strategies of resistance tend to prevail even when there is disconfirming information available to the conservative service managers.

A key feature of a 'learning organisation' is that people within the organisation, particularly management, are able to see the wider picture and how their own setting fits into this wider picture [23]. It seemed for those projects under consideration in this study that within NHS settings this was harder to achieve given the bureaucracy within and the hierarchical nature of the system.

### Outcome

Although this article is not reporting on the individual projects but focusing on cross-project lessons, it is worth noting two points about outcomes at the individual project level. First, some of the projects already had a proven track record of success and were 'good bets' for the funder, who was effectively funding an extension of that success. Indeed, by the end of the evaluation that confidence from the commissioners of those projects was well founded because the projects continued to demonstrate success.

Second, assessing the degree of individual project successes by the end of the evaluation was necessarily provisional. Some projects were ongoing and would end beyond the period of time agreed for the evaluation. All had been sustained and had demonstrated local impacts, but it was not clear what might happen to them in the long term (especially in a climate of resource constraints). For example, one NHS based project from the outset encountered some resource envy from managers of other sub-systems in the Trust involved. In an emerging context of budget capping and financial retrenchment, any long term commitment to the project by the employing Trust might require monies being lost elsewhere in the system.

Another consideration is about predicting the potential for 'spread,' where projects were demonstrated in one locality but then awaited testing elsewhere. This question of generalisability was not the same for all 11 attempts at innovation, because their aspirations for more general impacts were not identical. Broadly, these aspirations were of three types. First, some were demonstration projects that could or might be replicated elsewhere (for example, the theatre project in secure services or the educational projects involving service users). Second, some aimed to create purchasable products to be sold on to others (for example, DVDs). Third, some projects wanted to extend their influence in the NHS (for example, the web-based patient feedback project). Thus any long-term judgments about the success of innovations need to be conceptualised in terms of the type of impact generalisation desired.

The model presented above provides a rich agenda to consider by any service wanting to innovate or adopt innovations from elsewhere. In the first case of demonstration projects, an adopter would need to check their own local conditions to assess which conducive and impeding factors were extant, and what they could do to engineer the correct ratio of conducive to impeding factors. In the second case, they would need to make an assessment, within their financial constraints, about the cost-effectiveness of buying the product and ensuring its proper dissemination and utilisation. In the third case, the challenge is ensuring that influence is sustained and is adapted in relation to changing conditions.

In all three cases, because predictions are very difficult to make in open systems, innovations will only be sustained by leaders and managers developing a self-conscious and determined approach to organisational learning and the need to nurture adaptive organisations [24]. The latter refer to organisations that adapt to the conditions of their sustaining context. Organisational leaders in adaptive organisations are context-sensitive in their approach to the prospects of innovation.

## Conclusion

This article has summarised the main findings and lessons learned from an evaluation of 11 mental health innovation projects. The focus has not been on the degree of success of the individual projects, but on the production of a model of conducive and impeding factors evident and the lessons to be learned from that composite picture. In addition, the evaluation suggested the importance of a combination of studying innovation in relation to context, process, and outcomes.

As the results demonstrated, there appeared to be an imbalance between conducive and impeding factors, with a clear higher prevalence of conducive conditions both in terms of the volume of items and the number of occurrences. This, at least intuitively, appears at odds with other studies of innovation implementation. This imbalance is likely to be attributable to the fact that the innovations included in the evaluation were targeted innovations with a strong rationale and enthusiastic project champions. The balance that is normally observed with a shift towards impeding conditions may refer to 'top down' roll-out type changes. This is likely to have clear implications relating to any conclusions made relating to the spread of innovations. For example, the impeding factors identified within this study are likely to have greater salience in sites asked to adopt innovations designed and tested elsewhere.

## Conflict of interests

Anne Rogers is an Associate Editor of Implementation Science.

## Authors' contributions

HB was involved in the development of the project, carried out the interviews, participated in the analysis and report writing for the project, as well as being involved in drafting the manuscript. DP was the principal investigator and had input into the data collection, analysis, and report writing. He was also involved in critically revising the manuscript for academic coherence. AR was involved in the design of the project, collected data, was on the steering group of the project, and had input into the data analysis and report writing as well as critically revising the manuscript for intellectual content. All authors read and approved the final manuscript.

## Authors' information

HB (BSc, MRes) is a Research Associate within Health Sciences - Primary Care, Community Based Medicine at the University of Manchester. DP (BSc, MSc, MPsychol, PhD) is Professor of Mental Health Policy at University of Central Lancashire and Honorary Professor of Clinical Psychology, University of Liverpool. AR (BA, MSc, PhD) is Professor of the Sociology of Healthcare, NIHR School for Primary Care Research, Community Based Medicine at the University of Manchester.
